# Exploring Embodiment Through Choreographic Practice

**DOI:** 10.3389/fpsyg.2018.01920

**Published:** 2018-10-15

**Authors:** Angela Pickard

**Affiliations:** Music and Performing Arts, Canterbury Christ Church University, Canterbury, United Kingdom

**Keywords:** choreography, embodied, Bourdieu, *habitus*, identity, gender, practice-as-research

## Abstract

This pilot explored embodiment and gender representation through the lens of choreographic practice and sociology. The perspective derives from a comparative lack of status held by female (vs. male) choreographers in the United Kingdom. The pilot study specifically addresses how choreography itself embodies and perpetuates sociocultural values. This work is part of a larger, on-going ethnographic study into the social world(s) of choreography and choreographers. The method is a process of dance making called Sonnet that would expose habitual expectations of dance performances. The process aimed to heighten awareness of gender expectations and to challenge dancers and audience members to reflect on what they normally take for granted. Using Pierre Bourdieu’s critique and notion of *habitus* (embodiment), the study indicates perpetuating social hierarchy in dance training and practice. This is explored and framed from Bourdieu’s social and cultural perspective.

## Introduction

This pilot explored choreographic practice and sociology of embodiment. Choreography is creation, design, and structure of movement on/with physical bodies. Choreographic methods as a process of practice-based research are used as qualitative data. It is argued that choreographic practices and performances are the methods and embodiment of the choreographer and that dancers as inseparable methods and material (Sabisch, 2011). Choreographers and dancers are biographical, and autobiographical, signifying artistic, social, aesthetic and political values and cultural connections in practice. Pierre Bourdieu’s critique of the perpetuating social order, ‘rules of the game’ *habitus*, and embodied narratives (Bourdieu, 1990; Pickard, 2012, 2013, 2015), are considered to understand more about the social world of choreography.

Since 2009 there has been a debate in the United Kingdom asking questions as to why there are few female choreographers producing high profile work. In 2009 dance critic, Judith Mackrell suggested that the current field of choreography is dominated by men:

‘Dance has always been seen as the one art form where women weren’t just more visible than men, but were also in charge. From the pioneering contemporary choreography of Martha Graham, to The Royal Ballet founder Ninette de Valois, to the late, great Pina Bausch,…(now) the dance scene…looks distinctly alpha male’ (www.theguardian.com)

That same year, there was a debate asking why dance choreography appears to be dominated by males titled ‘*Where Are All The Women*?’

In 2013, Jennings discussed how fewer women achieve large-scale and high-profile commissions in the United Kingdom:

‘What the history of British classical dance overwhelmingly demonstrates is that while women may run ballet schools and become ballet company administrators and directors, they are rarely, if ever, invited to the choreographic high table. They are permitted responsibility, in other words, but not creative power.

The consequence in recent years has been a succession of works, some forgettable, some memorably fine, but all bearing a recognizably male creative stamp.’

Despite some shift in the profiles of female choreographers internationally, this debate is still a live one in the United Kingdom.

This pilot was an opportunity to begin to question the social world of choreography and taken-for-granted male dominance. This was from the perspective of a female artist making work with a female and male dancer, as well as audiences’ responses. In addition, the work that was created and performed was intended to offer a critique of traditional roles and relationships. This study was the starting point to a larger, longitudinal study exploring experiences and embodied identities of female choreographers in the United Kingdom and internationally.

## Bourdieu

Pierre Bourdieu’s conceptual schema was used to make more sense of social practice and agency as *logic of practice* (1990). Bourdieu developed the concept of *habitus,* as systems of habits and dispositions that become taken-for-granted and part of everyday life. He linked agency (practice) with structure, via capital or resources, and field, as socially structured conditions or context. A field is a context or network for power; an increase in capital is currency. Currency can be physical, social, or cultural for example. Currency enables agents to compete for power. Fields are like games that individuals play but an understanding of ‘rules of the game’ is needed in order to compete for power.

The process of *habitus* shapes actions and reproduces the field. For example, in my work with young ballet dancers (Pickard, 2012, 2013, 2015), learning to deal with pain was accepted and taken-for-granted as a core part of the *habitus* and embodied identity within schooling. *Habitus* is the way the social game is inscribed in individuals as a *logic of practice* (Bourdieu, 1990). *Habitus* generates practices as a perpetuating social order because it is unreflective socialization. It is suggested that the perpetuating social order in the United Kingdom is that choreographers tend to be male.

## Methods

The project took place across one academic year and adopted an ethnographic approach (Hammersley and Atkinson, 2007). At mid-point in the process a number of academics from a range of disciplines were invited to respond to the choreographic work (Lerman, 2003) as a form of triangulation. The dance work ‘*Sonnet*’ was performed on three occasions in Canterbury and Edinburgh, United Kingdom. Audiences were varied in age, nationality, race, and experience of watching dance. 143 audience members in total, from the three venues, completed a questionnaire. Qualitative comments as reflexivity (Hammersley and Atkinson, 2007) were noted and filmed during the process of making.

One female dancer, 20 years old, one male, 25 years old, and a female musician (saxophonist) were invited to participant in the project. The dancers were chosen due to their backgrounds as a classical female dancer and a contemporary male dancer. Appropriate ethical clearance and risk assessments were undertaken. Pseudonyms have been used in this paper.

## Findings and Discussion

Data from the dancers and audience members were used to begin to illustrate *habitus*, capital, ‘rules of the game’ and embodied narratives.

### Perceptions of the Dancers

The female dancer, Amy, summarized her *habitus* as a classically trained ballet dancer:

‘From a young age I have been encouraged to look a certain way, act a certain way and be a certain way. As a female dancer I have to be perfect, feminine and more submissive to male dancers who tend to lead and control.’

This is a useful illustration of norms and expectations associated with ballet aesthetic, *habitus* of ballet training and bodily *hexis* (Bourdieu, 1990). Hierarchical gender relations are embedded in bodily *hexis* as embodied actions that structure how a person thinks, feels and acts as ingrained in an individual’s psyche and body; actions become habitual, intuitive, and subconscious (Pickard, 2015, p. 13).

Amy discussed expectations and barriers:

*‘I think you have to be prepared to do anything for a choreographer and not complain as they are in charge.* *It is more competitive being a girl as there are less boys so they automatically get roles whereas we have to audition.* *I don’t see myself as a choreographer as I do, probably wrongly, associate choreographers as men. If you want to be a choreographer or a dancer actually, then you have to be confident to go for it early as if/when you have children, I think childcare is a problem. You’d need a husband who has flexible working, family who could help out, a nanny or a company that can function without you as choreographer on tour, so a good rehearsal director. I think this might put women off trying to be a choreographer.’*

This view was supported by Jennings (2013),

‘In classical dance, female choreographers are rare indeed, and the dynamics of vocational ballet schooling are at least partly responsible. Boys see themselves as individuals from the start, but girls quickly learn how replaceable they are, and in consequence can become over-anxious to ‘fit in” (www.theguardian.com).

Jennings (*ibid*) continued:

‘any girl dreaming of a career in choreography had better mind the glass ceiling…“When I was a student,” one Royal Ballet soloist remembers, ”the highest praise was to be told that you were a ’good girl’.” While this makes for loyal, biddable corps de ballet dancers, it doesn’t encourage young women to take a proactive approach to their own creative careers.’

The male dancer, Finn, suggested that male choreographers have greater confidence/capital to broker larger venues:

‘There are more male than female choreographers I would say. I think once a boy enters dance and is able to commit he is encouraged to be ambitious as few boys stick with it because of prejudice. Males are more confident to approach larger venues and get commissions, whereas I think females may try for smaller places.’

Finn continued:

‘Often a male choreographer is more physical and expects the men in the work to be athletic, and show-off. I think this is appealing to an audience. The female choreographers I know, apart from Jasmin Vardimon, tend to choreograph smaller, more somatic works, and work in the independent dance sector.’

Bodies of males are often choreographed and performed in hyper-masculine ways (Burt, 1995; Gard, 2001, 2006; Risner, 2007) as powerful and athletic. Males tend to use large, expansive movements and space as *performativity* of gender (Butler, 1994). Capital is gained through demonstration of physicality and risk. As Finn discussed:

‘I think men are encouraged to break rules, from a young age, in education but also in dance training. It’s manly to do this, take a risk. Risks mean creativity to me. I would say girls are taught to be good if you like, not take risks for fear of getting told off, do not ask questions or take risks but take direction. I would say this means they are taught to be dancers not choreographers.’

### Sonnet

The choreography *Sonnet* offered opportunities for the dancers to reflect on taken-for-granted ‘rules of the game’ (Bourdieu, 1990). *Sonnet* is intended to portray beauty, love, pain and loss in its lyrical quality, particularly from the perspective of the male dancer. The study was designed to investigate the dancers’ impressions of the process of learning and performing the dance as well as survey the audience responses.

Sonnet shows a reversal of traditional roles in a duet. The female dancer was given more opportunity to lead and material that demonstrated physicality, athleticism and a greater use of space. This was seen as liberating as well as challenging for Amy.

‘I was tentative as I am used to being led. The improvisation tasks were also challenging as I was making more decisions in terms of the speed and space aspects as well as the movement. The material I was given was also more technically underpinning and physical. My ‘character’ was also harsher than I think I would be in this situation so it was difficult to portray this. By the end of the work I was more confident and enjoyed the process. I have greater confidence to subvert tradition and take more risks. I like choreographing but tend to play safe and do more predictable things.’

Heterosexual cultural frameworks demand that males perform as subjects in positions of control, often to justify their decision to dance. To overtly express emotions is often associated as feminine. The male dancer, Finn, spoke about the opportunities he had in *Sonnet* to explore the wider possibilities of the male physical body:

‘Participating in this work has made me think more about the possibilities in expressing emotion through smaller movements and details. I have thought more about gender roles and the power of the female as dominant and controlling. I, as the more emotional. It has made me reflect on this as it has been a challenge to portray being submissive and not leading the duets. I enjoyed this though as the challenge was in being more submissive.’

### Audience Responses

It is suggested that dance performance is immersive for an audience and that each audience member can make psychological, physical, and social connections (Sheets-Johnstone, 2009). This is dependent on the audience values, expectations and beliefs. *Sonnet* is a dance duet inspired by Shakespeare’s poetic work. It uses live saxophone and the structure of the iambic pentameter and form of meter as phrasing in movement. At first, the work appears to offer traditional roles and relationships, as very typical and classical in form, but that which the dancers portray is intended to be opposite. The choreography illustrates complexities in *habitus* (Bourdieu, 1990), with the male and female duo dancing altering views on gender expectations. Throughout the piece, the male is gentle and withdrawn whilst the female is evidently the more powerful and confident.

The audience members did not know that the author was seeking comments on gender representation but these arose as a result of the choreography and dancers’ performances. One audience member said:

‘I loved this piece, a simple set, saxophone and a duet. I was surprised by the change in gender roles and enjoyed seeing the more emotional, lyrical movements from the male dancer and the strong, with more physical height and complexity from the female. I liked the way she manipulated and controlled him. It did make me think as it is different to the usual duets.’

Furthermore, another audience member responded:

‘I think we need more work like this – that blurs boundaries of gender. Why not have more, strong, female characters leading duets? Often they are stereotyped in classical work, with the male leading the submissive female. You can tell this work was made by a woman as there was greater emphasis on the female ‘character’ in the piece and the Sax player was female too – a conscious choice I don’t doubt.’

In addition, the same audience member thought there was more potential:

‘I was not fully convinced that the female was always leading. She seemed tentative at times and I would have liked more tension but also more strength and dynamics from her. I think with more immersion, she could find her more forceful side. You can tell she is a classically trained dancer and probably not used to leading. I’m used to watching/expecting the man to lead so I was consciously waiting for this to happen.’

### Reading Male and Female Bodies

Choreography can represent bodies as traditional by prescribing clear definitions and expectations of gender in performance, with set roles and behaviors for female and male dancers. Or choreography can offer a subversion. The body is a site of production and reproduction of particular cultural norms and gendered identity, or as Bourdieu argues, ‘the body is in the social world but the social world is also in the body’ (1990, p. 190). This was the premise for examination:

*‘each moment of watching a dance can be read as the product of choices, inherited, selected or invented about what kinds of bodies and subjects are being constructed and what kinds of arguments about these bodies and subjects are being put forth’* (Foster, 2011, p. 4).

Decades of choreographic work from male perspectives may be conditioning audiences to expect certain physicality and structure in work, as legitimate practice and perpetuating social order.

The process and performances in this pilot project were lived, constructed, embodied and narrated by the choreographer and dancers. *Habitus* is produced through beliefs about the body and conveyed through the repetitive structures of society as ‘logic of practice’ (Bourdieu, 1990). This project enabled a questioning of taken-for-granted ‘rules of the game.’ Although the study only has data from the two dancers and 143 audience members, the work has enabled some critical engagement in the process of making, gendered roles and expectations, and in understanding choreography as embodied performance.

## Conclusion

The intention of this pilot was to work intensively as a choreographer with two dancers in order to begin to critically engage with what might be ‘taken-for-granted’ expectations in dance. The data from audience members has revealed some patterns and trends that begin to evidence the initial assumptions around the status of female choreographers. The data is still being analyzed and will form the basis for longitudinal work.

This practice-based study used Bourdieu’s theoretical contribution as a way of understanding the processes via which choreography and dancer identities are constructed and perpetuated. Findings reveal relationships between the dancers, the social worlds of dance and choreography as gendered and embodied performance. In 2009, conclusions from the ‘*Where Are All The Women*?’ debate suggested that there should be access to greater networks and initiatives, such as prizes and platforms for female choreographers. In 2012, a £600,000 grant for new work at Sadler’s Wells theater was awarded to a female choreographer Liv Lorent. In 2015, artistic director Tamara Rojo commissioned a triple bill of works by three female choreographers entitled *She Said*. Contemporary dance culture does have more women working as choreographers and some are developing a high profile such as Jasmin Vardimon.

There may be positive moves toward improving the status of female choreographers. However, much can be done to build expectations that value female choreographers in the United Kingdom. Embodiment can be viewed as an unfinished entity in which bodies have the potential to change and transform. If more female choreographers were given platforms to share work with wider audiences, this may provide the context for an altered framework of embodiment.

## Ethics Statement

Canterbury Christ Church University Consent via information letter and consent form.

## Author Contributions

The author confirms being the sole contributor of this work and has approved it for publication.

## Conflict of Interest Statement

The author declares that the research was conducted in the absence of any commercial or financial relationships that could be construed as a potential conflict of interest.
